# PET Imaging of Acidic Tumor Environment With ^89^Zr-labeled pHLIP Probes

**DOI:** 10.3389/fonc.2022.882541

**Published:** 2022-05-19

**Authors:** David Bauer, Hannah Visca, Anuradha Weerakkody, Lukas M. Carter, Zachary Samuels, Spencer Kaminsky, Oleg A. Andreev, Yana K. Reshetnyak, Jason S. Lewis

**Affiliations:** ^1^ Department of Radiology and the Molecular Pharmacology Program, Memorial Sloan Kettering Cancer Center, New York, NY, United States; ^2^ Department of Physics, University of Rhode Island, Kingston, RI, United States; ^3^ Department of Medical Physics, Memorial Sloan Kettering Cancer Center, New York, NY, United States; ^4^ Department of Radiology, Weill Cornell Medical College, New York, NY, United States; ^5^ Department of Pharmacology Program, Weill Cornell Medical College, New York, NY, United States

**Keywords:** pH-low insertion peptides, membrane-insertion behavior, acidic tumor microenvironment, zirconium-89, PET imaging, human dosimetry estimates

## Abstract

Acidosis of the tumor microenvironment is a hallmark of tumor progression and has emerged as an essential biomarker for cancer diagnosis, prognosis, and evaluation of treatment response. A tool for quantitatively visualizing the acidic tumor environment could significantly advance our understanding of the behavior of aggressive tumors, improving patient management and outcomes. ^89^Zr-labeled pH-low insertion peptides (pHLIP) are a class of radiopharmaceutical imaging probes for the *in vivo* analysis of acidic tumor microenvironments *via* positron emission tomography (PET). Their unique structure allows them to sense and target acidic cancer cells. In contrast to traditional molecular imaging agents, pHLIP’s mechanism of action is pH-dependent and does not rely on the presence of tumor-specific molecular markers. In this study, one promising acidity-imaging PET probe ([^89^Zr]Zr-DFO-Cys-Var3) was identified as a candidate for clinical translation.

## Introduction

Cancer is a complex disease with potentially high heterogeneity between tumors and within an individual tumor and its metastases (1). Tumor growth and progression depend not only on tumor genotype but also on the metabolic status of cancer and the immune cells within the tumor microenvironment (TME). Cancer cells alter their metabolism (metabolic switch) to support their rapid proliferation and dissemination across the body, manifested in high rates of glucose consumption and an overexpression of surface carbonic anhydrases (e.g., CA IX), which catalyze the transformation of carbon dioxide and water into carbonic acid (2). As a result of anaerobic (3) and aerobic glycolysis (4) (Warburg Effect) and the overexpression of carbonic anhydrases, cancer cells contribute to the acidification of the TME. Also contributing to acidity are tumor-associated macrophages (TAMs). The progression of immune-excluded (“cold”) tumors is associated with the presence of acidic metabolically active TAMs, which generate immuno-suppressive signals, enhance angiogenesis, and promote metastases (5, 6). A better understanding of these processes could greatly impact patient outcomes.

**Figure 1:**
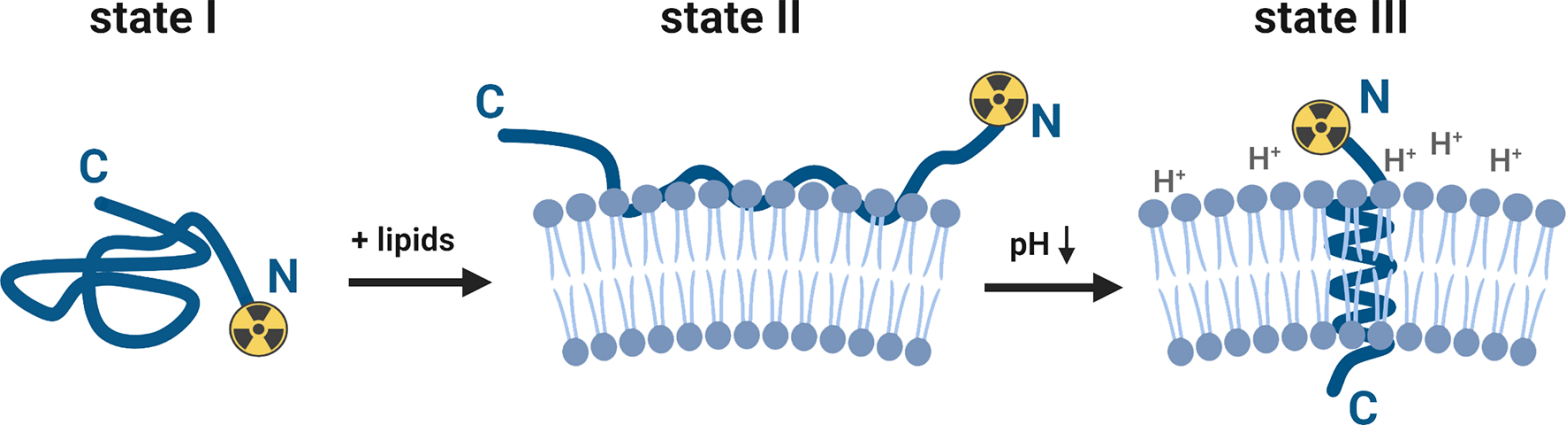
Schematic presentation of the interaction of a pHLIP-cargo conjugate with the lipid bilayer of a membrane at neutral/high and low pH values (for purpose of this study, the cargo represents a [^89^Zr]zirconium-chelate, attached to the N-terminus). The pHLIP sequence is an unstructured coil in the solution at neutral and high pH values (state I). The equilibrium shifts towards the membrane-bound state II when lipids (e.g., cell membranes) are added to the system. At a low surface pH (6.0–6.2), aspartic acid and glutamic acid residues are protonated and the overall hydrophobicity increases. This triggers the partitioning of the peptide into the lipid bilayer. The equilibrium at low pH values is shifted towards the membrane-inserted state III, which is accompanied by membrane-associated folding to form a transmembrane alpha-helix. This figure was created with BioRender.com.

The ability of the pHLIP family to sense and target acidic cancer cells and TAMs within the TME could be leveraged to investigate tumor biology and predict cancer treatment responses. As such, pHLIP technology is a highly active area of research (see reference for a comprehensive review) (7). Briefly, pHLIP is a water-soluble unstructured peptide at neutral and high pH values (state I, **Figure 1**). Being a moderately hydrophobic peptide, pHLIP exhibits a high affinity for the cell membrane (state II, **Figure 1**). Several carboxyl groups within the pHLIP sequence are protonated at a low cell surface pH, which triggers the peptide’s folding and insertion across the cell membrane to form a stable transmembrane helix (state III, **Figure 1**). The dielectric environment at the membrane slightly increases the pKa values of the carboxyl groups. At low local pH values (6.0–6.5), found at the surface of metabolically active cells (8–10), the environment promotes the peptide’s protonation. Variation and truncation of the original pHLIP sequences allowed us to identify pHLIP Var3 as the lead candidate for clinical translation because of its optimal pK values and improved insertion rates as well as suitable pharmacokinetic and pharmacodynamic properties (11, 12). A variety of imaging and therapeutic agents have been successfully delivered by pHLIP agents to tumors (in more than 20 different human and murine tumor models). The cargo (payload) is attached either to the N-terminus of the peptide — the end that remains in the extracellular space (**Figure 1**) — or to the membrane-inserting end (C-terminus) (13–29). The pHLIP’s tumor uptake correlates with the tumor’s extracellular pH (30–32) and can be enhanced by acidification using co-injection of glucose (33) and overexpression of CA IX (31). In addition to primary tumors, satellites near the primary tumor and micro-metastases in distant organs are targeted by pHLIP agents (33–36). It was also demonstrated that pHLIP conjugates target the acidic TAMs within the TME (28).

The acidic TME can be imaged with pHLIP-based PET-compatible radiotracers. Clinically, a pHLIP-based radiotracer could provide more information about the TME and tumor progression than a standard [^18^F]FDG PET scan. Our groups have evaluated ^18^F-labeled (37), ^99m^Tc-labeled (32), ^64^Cu- and [^18^F]AlF-labeled (38), as well as ^68/67^Ga-labeled (39) pHLIP analogues in various tumor models. A phase I clinical trial on breast cancer with an investigational ^18^F-labeled pHLIP probe (based on a d-amino acid sequence of Var3 pHLIP) was completed at Memorial Sloan Kettering Cancer Center (MSK) (NCT04054986). The phase I protocol was performed as a first-in-human PET/CT trial of five patients with metastatic breast cancer and demonstrated the safety and slow blood clearance (several hours) of the ^18^F-labeled pHLIP conjugate. The short half-life and/or availability of the radionuclides mentioned above is a significant limiting factor for long-term circulating pHLIP compounds. In preclinical PET imaging, an optimal tumor contrast has been observed with pHLIP at or after 24 hours. For this reason, long-lived PET radionuclides, such as the widely available zirconium-89 (^89^Zr), would likely maximize clinical diagnostic potential with pHLIP. Here, we investigated several ^89^Zr-radiolabeled pHLIP imaging agents with the goal of introducing a novel PET pH-sensor with optimized pharmacokinetics and a high tumor uptake for a possible clinical translation.

## Materials and Methods

### pHLIP Conjugates

The d-amino acid versions of the Var3 and WT pHLIPs were synthesized and conjugated with the chelators by the company CS Bio (Menlo Park, CA) with ≥ 95% purity. The HOPO chelator was provided by the laboratory of Dr. Lynn Francesconi (Chemistry Department at Hunter College, New York). The DFOsqa chelator was synthesized following a procedure described in the literature (40). ^1^H- and, ^13^C-NMR (nuclear magnetic resonance), and ESI MS (electrospray ionization mass spectrometry) analysis did match the literature records [SI (40)]. All other chemicals were purchased from commercial suppliers without further purification unless otherwise stated.

### Radiochemistry

[^89^Zr]Zr-oxalate in 1 M oxalic acid was received from 3D Imaging LLC (Little Rock, AR). All activities recorded in this study were determined by an Capintec^®^ CRC-55tR dose calibrator. For the radiolabeling, the required activity (185 MBq/5 mCi) was transferred into a Protein LoBind^®^ Eppendorf tube and adjusted with 1 M HEPES [4-(2-hydroxyethyl)-1-piperazineethanesulfonic acid] buffer [four-times the volume of (^89^Zr)Zr-oxalate] to pH 7.0–7.4. To this, the pHLIP conjugate was added [60 µL of a 5×10^-4^ M pHLIP-solution in DMSO (dimethyl sulfoxide), equivalent to 30 nmol].

The mixture was incubated at 37°C for 30 minutes. The completion of the radiolabeling was checked by radio instant thin-layer chromatography (iTLC) using iTLC-SG chromatography paper (Agilent Technologies) and an aqueous EDTA (ethylenediaminetetraacetic acid) solution (50 mM, pH 4). The iTLCs were scanned on an radio-TLC imaging scanner (AR2000; Eckert & Ziegler Radiopharma GmbH). The radiochemical yield of all constructs exceeded 95%, and they were used without purification. The ^89^Zr-radiolabeled pHLIP were prepared for intravenous injection by diluting the reaction mixture with sterile filtered phosphate-buffered saline (PBS) to the required volume (2.5 mL). Each mouse received 100 µL of PBS solution containing 1.2 nmol of pHLIP and 7.4 MBq/200 µCi of activity.

A potential degradation of the compounds was investigated by serum stability assay. For this, 100 µL of the prepared PBS solution (containing 1.2 nmol of pHLIP and 7.4 MBq/200 µCi) was added to 900 µL of human serum (heat inactivated from Millipore Sigma) and incubated for seven days at 37°C. The release of free ^89^Zr^4+^ was measured by radio instant thin-layer chromatography (as described above) and the degradation of the whole construct was checked by radio high-performance liquid chromatography (HPLC). The analytical reverse-phase HPLC was performed on a Shimadzu system equipped with a Flow Count PIN diode radiodetector from BioScan, a DGU-20A degasser, two LC-20AB pumps, and an SPD-M20A photodiode array detector. A BetaBasic 18 column (150 Å, 5.0 µm, 4.6×150 mm, Thermo Scientific) was used with water (+ 0.1% trifluoroacetic acid) and acetonitrile (+ 0.1% trifluoroacetic acid) as solvents. The gradient started at 5% acetonitrile and increased to 95% over 15 minutes at a constant flowrate of 1 mL/min. The radiochemical purities of all ^89^Zr-radiolabeled pHLIP conjugates were greater than 90% over the course of seven days.

### Biophysical Studies

The interaction of the pHLIP agents with liposomes was investigated by recording the tryptophan fluorescence and circular dichroism (CD) spectra by using a PC1 spectrofluorometer (ISS, Inc) and an MOS-450 spectrometer (Biologic, Inc), respectively, with temperature control set to 25.0°C. Liposomes, consisting of large unilamellar vesicles, were prepared by extrusion. POPC (1-palmitoyl-2-oleoyl-*sn*-glycero-3-phosphocholine) (Avanti Polar Lipids, Inc.) in chloroform was desolvated on a rotary evaporator and dried under vacuum for several hours. The phospholipid film was rehydrated in 10 mM phosphate buffer, pH 8.0, vortexed, and passed 21 times through the extruder (50 nm membrane).

Using an excitation wavelength of 295 nm, tryptophan fluorescence spectra were recorded from 310–400 nm. CD spectra were recorded from 190–260 nm with 1-nm steps. The concentration of the peptide and the POPC liposomes varied in different experiments: 5–15 μM of pHLIP agents and 1 mM of POPC liposomes.

The pH-dependent insertion of the peptides into the lipid bilayer of the POPC liposomes was studied by monitoring either the changes in tryptophan fluorescence spectra or changes in the molar ellipticity at 222 nm as a function of the pH value. After the addition of aliquots of HCl, the pH values of the solutions containing peptide and POPC liposomes were measured using an Orion PerHecT ROSS Combination pH Micro Electrode and an Orion Dual Star pH and ISE Benchtop Meter. Fluorescence spectra were analyzed using the Protein Fluorescence and Structural Tool Kit (PFAST) (41) to determine the positions of spectral maxima (λ*_max_*). The λ*_max_* and millidegree data were normalized to 0–1 and were plotted as a function of pH. The pH-dependence was fit with the Henderson-Hasselbach equation to determine the cooperativity (*n*) and the mid-point (*pK*) of transition:


$$Normalized\;pH\;dependence=\frac{1}{1+10^{n\left(pH-pK\right)}}$$

The tryptophan fluorescence kinetics were measured using an SFM-300 mixing system (Bio-Logic Science Instruments) in combination with the MOS-450 spectrometer with temperature control set to 25.0°C. All samples were degassed before the measurements to minimize air bubbles in the samples. The peptide and POPC samples were incubated overnight to reach equilibrium, to assure that most of the peptide is associated with the liposome lipid bilayers. To follow the peptide insertion, equal volumes of the peptide-POPC solution and of HCl were mixed to lower the pH from 8 to 4. To monitor fluorescence intensity changes during the peptide insertion into POPC liposomes induced by the pH drop, the tryptophan emission signal was observed through a cut off 320 nm filter at an excitation of 295 nm.

All data was fit to the appropriate equations by nonlinear least squares curve fitting procedures employing the Levenberg Marquardt algorithm using Origin 8.5.

### Cell Preparation and Animal Models

The RM-1 and 4T1 cell lines were purchased from ATCC and cultured according to the recommended conditions at 5% CO_2_ atmosphere and 37°C in DMEM (Dulbecco’s Modified Eagle Medium) and RPMI 1640 medium, respectively, each containing 10% fetal bovine serum. The media were provided by the MSK Media Preparation Core. For the subcutaneous allografts, the cells were stripped in the absence of magnesium or calcium ions using a mixture of 0.25% trypsin and 0.53 mM EDTA in Hank’s Balanced Salt Solution, concentrated in 1 mL of the corresponding medium, and a small aliquot was used to determine the cell count (Beckman Coulter Vi-CELL XR). Another aliquot was diluted with the medium so that 50 µL contained 1×10^6^ cells.

The imaging and biodistribution studies were performed with male (RM-1 model) and female (4T1 model) athymic nude mice and the kidney-blocking experiments were performed with female SCID mice received from Charles River Laboratories (Stone Ridge, NY). After arrival, the mice were kept in the MSK vivarium for one week before any experimental handling was performed. The animals were allowed free access to water and food, and all animal care and experimental procedures were approved by the Institutional Animal Care and Use Committee (IACUC). For the subcutaneous allografts, a 1-to-1 ratio of Corning Matrigel Matrix and the cell solution was prepared and stored on ice for the injections. Each mouse received a subcutaneous injection of 100 µL (50 µL cell solution, viability > 90%, + 50 µL Matrigel) into the flank on the level of the right shoulder. The injections were performed under anesthesia (2% isoflurane, Baxter Healthcare, Deerfield, IL, USA). Palpable tumors of similar size (100–300 mm^3^) developed 5–8 days after grafting.

### Small Animal *in vivo* PET Imaging and Post-Mortem Biodistribution Studies

The mice were intravenously injected (tail vein) with the radiolabeled pHLIP conjugates (1.2 nmol, 7.4 MBq/200 µCi, 100 µL, in sterile filtered PBS). The activity of the syringe prior to injection and after injection was used to determine the percent of injectate administered.

PET images were obtained at the respective timepoints with the mice under anesthesia (2% isoflurane) on a microPET Focus 120 (Concorde Microsystems) or an Inveon PET-CT (Siemens) rodent scanner. All images were analyzed using the Medical Imaging Data Examiner Amide (version 0.9.0) or the Inveon reconstruction software suite. The counting rates in the reconstructed images were converted to percent of injected dose per weight (%ID/g) by applying a system-specific calibration factor. At the final timepoint, the remaining mice were euthanized (by CO_2_ asphyxiation, followed by cervical dislocation) and their tumors were resected for histological analysis.

For the biodistribution studies the tumor-bearing mice were randomized before the injections. Only mice with suitable tumors (150–300 mm^3^) were utilized. An exception was made for the 120-hour timepoint, for which only mice with smaller tumors (150 mm^3^) were selected. The mice were grouped in cohorts of four. At the respective timepoint, the mice were euthanized (by CO_2_ asphyxiation, followed by cervical dislocation) and their blood was collected by cardiac puncture as a terminal procedure, followed by quick removal of the tumors. After this, the other organs/samples were collected. All samples were weighed and the radioactivity for each one was counted for 1 minute using an automatic gamma counter (Wizard^2^ 2480 3′′, PerkinElmer, Waltham, MA), resulting in less than 10% error due to counting statistics for each measurement. The exact activities of all samples of one mouse were determined by an Capintec^®^ CRC-55tR dose calibrator. These decay-corrected measurements were used to determine a calibration factor and calibrate all counts to the corresponding activities. Activity concentrations were calculated as percentage injected dose per tissue weight (%ID/g wet tissue). All data was processed and visualized using Origin 8.5.

The animal studies, including the different radiotracers and animal models, are listed in **Table S1**.

### Dosimetry

Briefly, human dosimetry estimates were extrapolated from the serial biodistribution and PET image data for [^89^Zr]Zr-DFO-Cys-Var3 in female nude mice. The decay-corrected percentage of administered activity in human organ *i*, %ID*_i,human_*, was calculated using the following equation, which assumes interspecies equivalence of the organ-level mean standardized uptake values (42):


$$\%ID_{i,human}=\frac{\%ID_{i,mouse}}{m_{i,mouse\;}}\times \frac{m_{mouse}m_{i,human}}{m_{human}}$$

Here, %ID*_i,mouse_*/*m_i,mouse_* is the activity concentration in mouse organ *i* (see **Table S2**), *m_mouse_* is the mouse total body mass (approx. 25 g); *m_i,human_* and *m_human_* are the blood-inclusive source organ mass and total body mass, respectively, of the computational phantom used to represent the reference adult patient (43, 44). The activity concentration in the blood was assumed to be representative of the red bone marrow. For each organ, the %ID*_i,human_* vs. time was modeled using mono- or bi-exponential functions, which were fit to the data using weighted least-squares regression using the Microsoft Excel SOLVERSTAT statistics package (45). The weight of each observation was taken as the inverse of the variance (computed from the SDs given in **Table S2**). The time-integrated activity coefficient (46) for human organ *i*, ${\tilde{a}}_{i}$

was computed from the analytical expressions for the integrals of the fit functions; the standard error in each ${\tilde{a}}_{i}$ , $\sigma_{{\tilde{a}}_{i}}$ was computed *via* Gaussian error propagation of the standard errors in the fit parameters, taking the covariances into account. Finally, the absorbed dose and effective dose coefficients were computed with MIRDcalc software (47), using the ${\tilde{a}}_{i}$ and $\sigma_{{\tilde{a}}_{i}}$ as input. 3D-absorbed dose maps were generated using PARaDIM software (48). The results can be found in **Table S3**

### *Ex vivo* Autoradiography, Staining, and Microscopy

The mice of the imaging groups were euthanized at the final timepoint (72 hours, by CO_2_ asphyxiation, followed by cervical dislocation) and their tumors were resected, embedded in OCT compound (optimal cutting temperature compound) and frozen. The following day, a series of contiguous tumor sections of 10 μm thickness were cut and exposed to a phosphor-imaging plate (Fujifilm BAS-MS2325, Fuji Photo Film, Japan) for 24 hours at −20 °C and read using a Typhoon 8600 photographic film scanner (GE Healthcare). Digital images of radioactivity distribution at 50 μm resolution were obtained. The same sections were subsequently used for H&E (hematoxylin and eosin) and CD31 (platelet endothelial cell adhesion molecule-1, PECAM-1, highlighting blood vessels) staining. The staining and scanning/digitalizing of all slices was performed by the MSK Molecular Cytology Core Facility. Autoradiographic and histological images were registered using Adobe Photoshop CS3 software.

### Cherenkov and Fluorescence Imaging

For the side-by-side imaging study, five male RM-1 tumor-bearing athymic nude mice were injected with a 100 µL PBS-solution containing [^89^Zr]Zr-DFOsqa-Lys-Var3 (1.2 nmol of pHLIP and 7.4 MBq/200 µCi) and the fluorophore Alexa546-Cys-Var3 (5 nmol). Alexa546-Cys-Var3 was provided by the laboratory of Prof. Dr. Yana K. Reshetnyak. At 48 hours post-injection, the mice were sacrificed and the kidneys, livers and tumors were resected and imaged. Cerenkov luminescence imaging (recording time of 5 minutes) and fluorescence imaging (recording time of 1 minute) was performed with an IVIS Spectrum (PerkinElmer). Cerenkov luminescence was recorded in p s^–1^ cm^–2^ sr^–1^ and fluorescence in p s^–1^ cm^–4^ sr^–1^ µW^–1^.

## Results

### Preparing the pHLIP Conjugates

**Figure 2:**
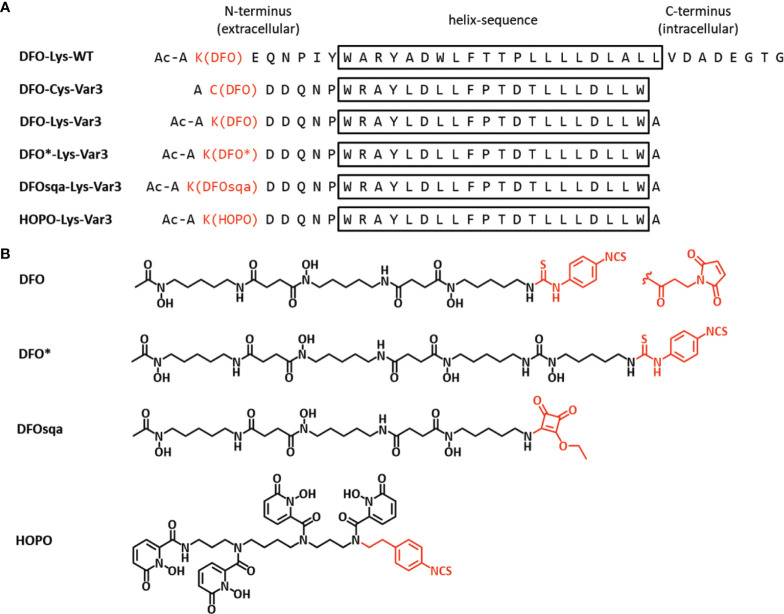
**(A)** The pHLIP sequences (d-amino acids) of the six investigated constructs in single letter code and **(B)** the chemical structures of the four chelators (active conjugation side in red). The conjugation was either performed by reacting the Lys-pHLIP with the isothiocyanate version of the chelator (activated ethyl ester for DFOsqa) or reacting the Cys-pHLIP with DFO-maleimide.

In this study, six ^89^Zr-labeled pHLIP conjugates were investigated. The pHLIP sequence was originally based on the wild type pHLIP (WT), which was isolated from the C-helix of the bacteriorhodopsin protein, a proton pump found in archaea organisms (13, 49). Optimization of this sequence led to Var3 pHLIP compound, which is used in this study (12). At the N-terminus, the pHLIP sequences can be modified with either a lysine (Lys-Var3 or Lys-WT) or a cysteine (Cys-Var3) residue, which can be used to conjugate a chelator by harvesting the cysteine–maleimide or a lysine–isothiocyanate reaction, respectively. The N-terminus of the pHLIP sequence remains in the extracellular matrix, while the C-terminus crosses the cell membrane into the intracellular space as shown in **Figure 1**. Previous studies confirmed that the cargo can affect the pharmacokinetics of the pHLIP conjugates (34); therefore, the choice of the chelator can be a critical factor. We investigated six ^89^Zr-labeled pHLIP conjugates (**Figure 2**) to identify the best candidate for a possible clinical translation. Five Var3 constructs were synthesized, using the zirconium-chelators DFO, DFO*, DFOsqa, and HOPO. The Zr^4+^-ion forms a +1 charged chelate with DFO and DFOsqa, while DFO* and HOPO form a neutral-charge chelate. It was previously shown that the WT pHLIP exhibits lower tumor targeting than the Var3 pHLIP. The WT sequence was used in this study to demonstrate the benefits of the optimized Var3 pHLIP version.

The radiolabeling of all pHLIP with [^89^Zr]zirconium oxalate conjugates resulted in a radiochemical yield > 95% (verified by iTLC and radio-HPLC) and no additional purification was necessary. The stability of the radiotracers was investigated in human serum at 37°C, indicating no release of unchelatssed ^89^Zr^4+^-ions or breakdown of the pHLIP constructs over a period of 7 days (**Figure S1**).

### Biophysical Characterization

**Figure 3:**
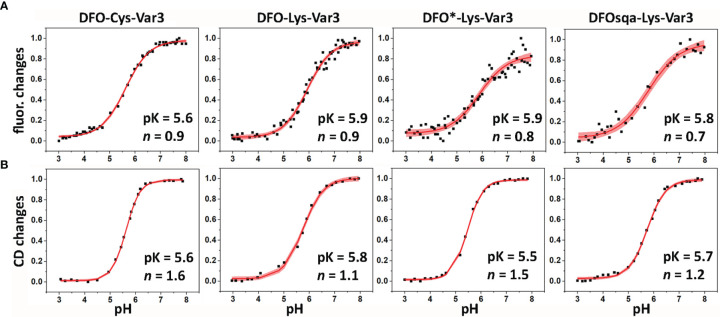
The pH-dependent insertion of pHLIP agents into the lipid bilayers of POPC liposomes was studied by monitoring the changes in the position of the maxima of the tryptophan fluorescence spectra **(A)** and ellipticity of the CD signals measured at 222 nm **(B)** as function of the pH. The data was fitted using the Henderson-Hasselbalch equation. The fitting curves (red lines) and 95% confidence intervals (pink areas) are shown.

A biophysical characterization of all pHLIP agents was performed to check on their membrane-insertion behavior. The pH-dependent bilayer interactions of the constructs were measured using POPC liposomes by changes in steady-state tryptophan fluorescence and CD. The tryptophan (Trp) fluorescence and CD was recorded for the pHLIPs in solution at a pH value of 8 (state I), and in presence of POPC liposomes at the pH of 8 (state II) and 4 (state III) (**Figures 1**, **S2, S3**, and **Table S4**). The fluorescence signal of the WT pHLIP cannot be directly compared with the emission of the Var3 constructs, since the Trp residues in the WT are located at the beginning and middle of the transmembrane helix, while the Trp residues in Var3 are located at the beginning and end of the transmembrane helix. Therefore, it is not surprising that the position of the fluorescence maximum of the DFO-Lys-WT in state III shows the lowest value (λ_max_ = 338.9 nm, **Table S4**). Among the five investigated Var3 agents, the spectral signals of the HOPO-Lys-Var3 were distinct, either because the HOPO-zirconium chelate absorbs light and/or its conjugation with pHLIP alters the pH-dependent behavior of the pHLIP. We also compared the spectral properties of the agents with and without chelated (natural) zirconium, and obtained very similar results (**Table S4**), indicating that the presence of the metal does not affect their spectral properties. Both DFO-Lys-WT and HOPO-Lys-Var3 were excluded from further biophysical studies. The pK value (midpoint of transition) and the cooperativity (*n*) of the peptides’ insertion into the membrane was investigated for the four remaining Var3 agents. The low wavelength shifts of the Trp fluorescence (fluor. changes, **Figure 3A**) indicate the peptide’s propagation into the hydrophobic environment of the lipid bilayer. The increase of their ellipticity (CD changes, **Figure 3B**) indicates a coil-helix transition. The normalized data fitted by the *Henderson*-*Hasselbalch* equation is shown in **Figure 3** and **Table S4**. All agents exhibit similar pK values with the highest cooperativity (*n* = 1.6) established for the DFO-Cys-Var3 coil-helix transition. The investigated agents demonstrated a fast insertion (msec) into the membrane (**Figure S4**) similar to the rate of the unmodified Var3 peptide (12). The influence of the metal-chelate complex on the peptide is minimal since it was attached to the membrane non-inserting end (N-terminus).

### *In vivo* Imaging Studies in RM-1 and 4T1 Tumor Models

**Figure 4:**
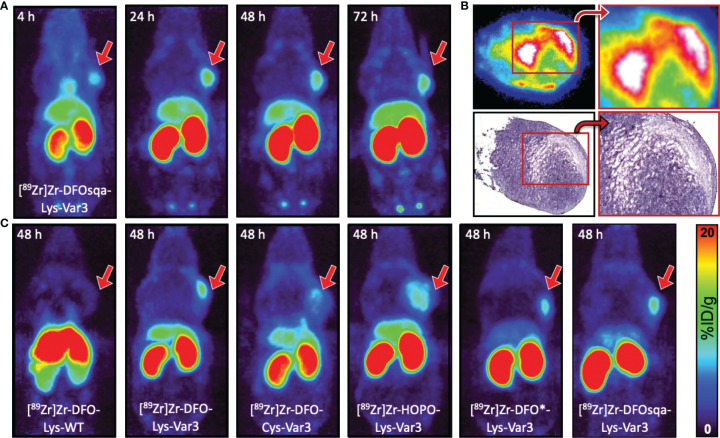
**(A)** Coronal MIP PET images at 4, 24, 48, and 72 hours of female athymic nude mice bearing subcutaneous 4T1 tumor allografts on the right shoulder, administered with 7.4 MBq/200 µCi of [^89^Zr]Zr-DFOsqa-Lys-Var3 (1.2 nmol pHLIP). **(B)** *Ex vivo* autoradiograph (top) and H&E staining (bottom) of 10 µm-tumor slices (excised at 72 hours post-injection), not scaled or calibrated. **(C)** Coronal MIP PET images at 48 hours of male athymic nude mice bearing subcutaneous RM-1 tumor allografts on the right shoulder, administered with 7.4 MBq/200 µCi and 1.2 nmol of the corresponding pHLIP conjugate. The scale bar applies to all PET images.

The murine cell lines RM-1 and 4T1, end-stage models for prostate and breast cancer, were respectively allografted subcutaneously into male and female athymic nude mice (1×10^6^ cells/mouse). Both cell lines grow rapidly; within one week of allografting, an optimal tumor size (100–300 mm^3^) was reached. Approximately 7.4 MBq/200 µCi (apparent molar activity of approx. 6.3 MBq/nmol) of the ^89^Zr-labeled pHLIP conjugate was intravenously administered (0.1 mL) to the tumor-bearing mice. PET scans were performed at 4, 24, 48, and 72 hours post-injection. The PET scans obtained from the 4T1 model, injected with [^89^Zr]Zr-DFOsqa-Lys-Var3, are displayed in **Figure 4A**. The variant for which serial biodistribution studies were performed, [^89^Zr]Zr-DFO-Cys-Var3, was cleared from the blood with a (biological) half-life of (16.0 ± 0.4) hours. A good tumor-background ratio for all compounds was observed for the 48-hour timepoint. All radiotracers were additionally evaluated in the RM-1 model and PET images at multiple timepoints were recorded. For all compounds, the 48-hour timepoint showed the best tumor-to-background ratio (**Figure 4C**); the tumor-to-muscle uptake-ratios ranged from 11.6 ± 4.9 to 16.9 ± 6.8 for the Var3 conjugates and was 5.5 ± 1.5 for DFO-Lys-WT. A comparison of the PET images between the 4T1 and RM-1 tumor models revealed a similar biodistribution of the constructs with slight variations in the tumor- and kidney-uptake (**Figure S5**). The radiotracers’ distributions are discussed in more detail in the “Biodistribution” section.

### Histological Tumor Analysis

After the 72-hour timepoint, all mice used in the imaging study were sacrificed. The tumors were resected, frozen in OCT compound, and sectioned (10 µm slices) the following day, followed by the recording of autoradiographs. Additional slides were prepared for H&E and CD31 staining (murine blood vessels staining). The pHLIP agents were present in the entire tumor mass, and the highest activity areas overlapped with the tumor stroma, as displayed in **Figures 4B** and **S6**, indicating the acidic hotspots within the TME. This result is in line with previously reported findings (50) showing that pHLIP compounds target the tumor–stroma interface, which serves as an acidic dump for cancer cells to maintain an optimal intracellular pH. A similar tumor distribution was observed for all pHLIP constructs. Additionally, 10 µm kidney sections were prepared, revealing that within kidney the ^89^Zr-labeled pHLIP compounds are present primarily in its cortex (**Figure S6**).

### Biodistribution Studies

**Figure 5:**
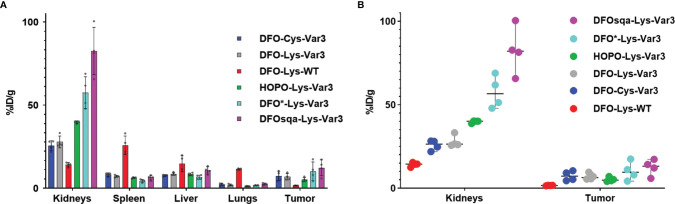
**(A)** Biodistribution data of the six investigated pHLIP constructs at 48 hours post-injection evaluated in the RM-1 tumor model (n=4). **(B)** Direct comparison of the kidney and tumor uptake.

The *in vivo* PET study revealed optimal tumor uptake and tumor-to-background contrast at the 48-hour timepoint. For this reason, the 48-hour timepoint was chosen for a single timepoint biodistribution study to compare the six pHLIP constructs more thoroughly in the RM-1 tumor model. The biodistribution data of the organs with the highest uptake is shown in **Figure 5A**. Additional data can be found in **Table S5**. All Var3 agents demonstrate similar biodistribution. This differs from the biodistribution of the WT agent, which demonstrates the lowest tumor targeting and highest spleen, liver, and lungs uptake. All Var3 agents exhibit high tumor uptake with the highest value (12.4 ± 4.7) %ID/g observed for [^89^Zr]Zr-DFOsqa-Lys-Var3. However, the high tumor uptake also correlates with a high kidney uptake, (82.5 ± 14.2) %ID/g for DFOsqa-Lys-Var3. The tumor-kidney-uptake correlation of all compounds is visualized in **Figure 5B**. It is reported that a significant pH gradient exists within the kidney parenchyma, related to the metabolic activity of the thick ascending limb of the loop of Henle, which might be of relevance for the acid-based homeostasis (51). This suggests that the radiotracers with a higher tumor uptake would also show an increased kidney accumulation.

### Cherenkov and Fluorescence Imaging

**Figure 6:**
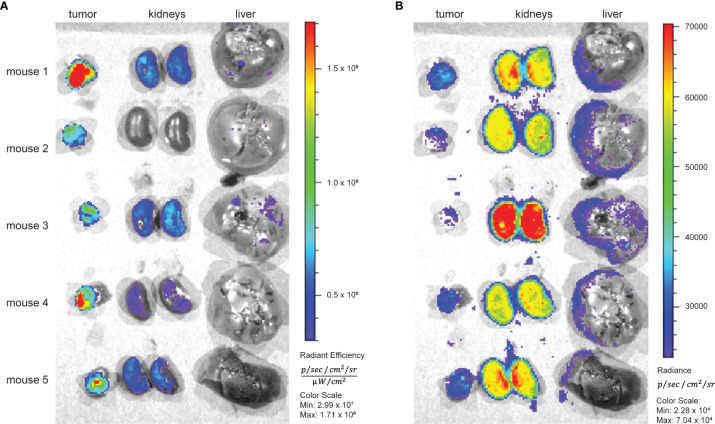
Five RM-1 tumor bearing male nude mice received 7.4 MBq/200 µCi [^89^Zr]Zr-DFOsqa-Lys-Var3 (1.2 nmol) and Alexa-546-Cys-Var3 (5 nmol) in a co-injection. At 48 hours the tumors kidneys and livers were resected, and fluorescence **(A)** and Cherenkov **(B)** images were recorded using the IVIS system.

The distinct kidney uptake was observed for all ^89^Zr-labeled Var3 conjugates. At the same time, according to the literature, fluorescently labeled pHLIP conjugates exhibit significantly less kidney uptake (34, 52) than radiometal-chelate-containing pHLIPs. Therefore, we carried out a side-by-side imaging study. Five male RM-1 tumor-bearing athymic nude mice were co-injected with [^89^Zr]Zr-DFOsqa-Lys-Var3 and fluorophore Alexa546-Cys-Var3. The mice were sacrificed at 48 hours post-co-injection and the kidneys, livers, and tumors were resected and imaged. Cherenkov irradiation originating from the ^89^Zr-decay and the Alexa546 fluorescence was recorded (**Figure 6**). Cherenkov imaging (expressed as radiance) and fluorescence imaging (expressed as radiant efficiency) are two different modalities. The organ uptake should be compared within the same modality. A slightly higher tumor-to-kidney ratio of Alexa546-Cys-Var3 was observed compared to the ratio for [^89^Zr]Zr-DFOsqa-Lys-Var3 — indicating that the metal-chelator constructs exhibit higher kidney retention.

### Blocking the Kidney Uptake

Additional experiments were performed to investigate the influence of various kidney-uptake blockers and diuretics. Healthy female SCID mice received a “blocking agent” 30 minutes prior to the injection of [^89^Zr]Zr-DFOsqa-Lys-Var3 (7.4 MBq/200 µCi, 1.2 nmol). The goal was to achieve a significant kidney clearance (uptake < 80%ID/g), as determined by PET imaging. Five different drugs were investigated: amiloride (epithelial sodium channel inhibitor), 5-(N,N-dimethyl)amiloride (more potent amiloride analog), probenecid (inhibits kidney uptake of organic anions), chlorthalidone (inhibits sodium reabsorption), and acetazolamide (carbonic anhydrase inhibitor). However, none of these drugs led to a significant reduction of kidney uptake compared to the control mice, which did not receive any inhibitor. The experimental set-up can be found in **Table S6**.

### Biodistribution and Dosimetry of the [^89^Zr]Zr-DFO-Cys-Var3 Lead Candidate

Due to elevated uptake, the kidney was expected to be the organ limiting administered activity for clinical PET. Since none of the kidney blocking strategies were effective in reducing the kidney uptake of [^89^Zr]Zr-DFOsqa-Lys-Var3, the [^89^Zr]Zr-DFO-Cys-Var3 agent, which shows (7.3 ± 2.9) %ID/g tumor uptake and (25.7 ± 3.0) %ID/g kidney uptake, was selected as the lead compound. A multiple-timepoint biodistribution was performed (4, 24, 48, 72, and 120 hours) using the 4T1 tumor model and [^89^Zr]Zr-DFO-Cys-Var3 (**Figure S7** and **Table S2**). Additionally, PET-CT images were recorded for each timepoint (**Figure S8**). The tumor- and kidney-uptake values [(9.7 ± 1.7) %ID/g and (47.6 ± 11.5) %ID/g, 48-hour timepoint] were significantly higher for this compound in the 4T1 model than in the RM-1 model [(7.3 ± 2.9) %ID/g and (25.7 ± 3.0) %ID/g, 48-hour timepoint].

Because the 4T1-tumors grew relatively quickly, smaller tumors (100 mm^3^) had to be chosen for the 120-hour timepoint, most likely leading to a slightly lower (not significant) tumor- and kidney-uptake for this timepoint [(6.8 ± 0.7) %ID/g and (35.3 ± 5.9) %ID/g, 120-hour timepoint]. Overall, the highest tumor uptake was detected for the 48-hour timepoint and a small but not significant tumor- and organ-clearance was observed for the 72- and 120-hour timepoints. A progressive drop in radiotracer blood level was observed over this five-day interval. A trace amount of (0.2 ± 0.05) %ID/g was detected for the 120-hour timepoint.

**Figure 7:**
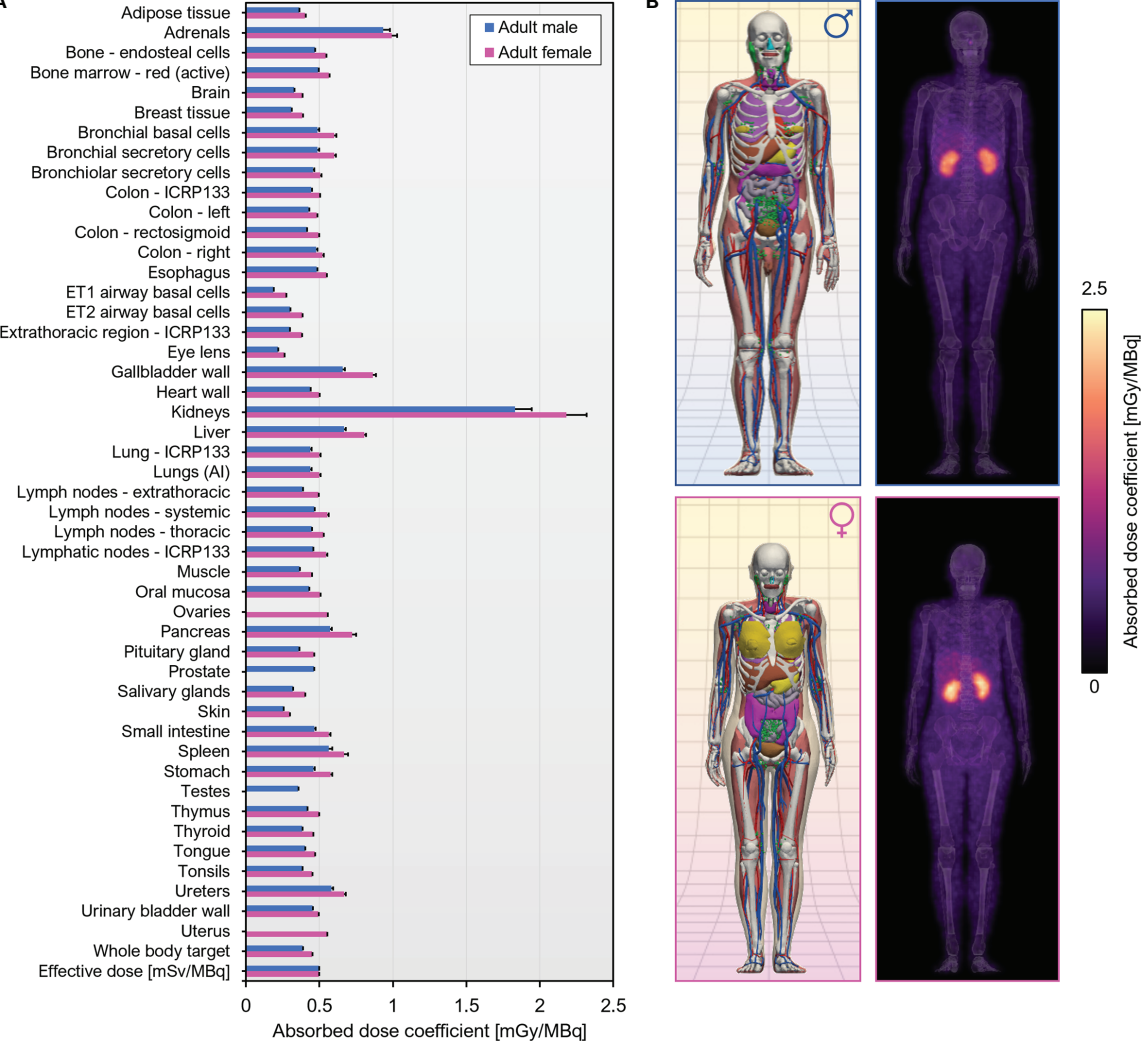
Human dosimetry estimates for [^89^Zr]Zr-DFO-Cys-Var3 projected from murine biodistribution. **(A)** Organ-level absorbed dose and effective dose coefficients computed for the ICRP 110 reference adult phantoms. Units are absorbed dose [mGy] per unit administered activity [MBq] unless otherwise specified. **(B)** Left, Computational phantoms used in dose calculations; Right, Maximum intensity projections of the 3D dose distribution.

Murine biodistribution data was extrapolated to reference human adults in order to obtain radiation dosimetry estimates for human i.v. administration of [^89^Zr]Zr-DFO-Cys-Var3. The effective dose coefficient was 0.50 mSv/MBq. The organs with the highest absorbed dose coefficients were the kidneys [male, (1.83 ± 0.11) mGy/MBq; female, (2.18 ± 0.14) mGy/MBq], followed by the adrenals [male, (0.934 ± 0.047) mGy/MBq; female, (0.991 ± 0.038) mGy/MBq] A graphical summary of the dose coefficients is given in **Figure 7**; tabulated values can be found in **Table S3**.

## Discussion

**Figure 8:**
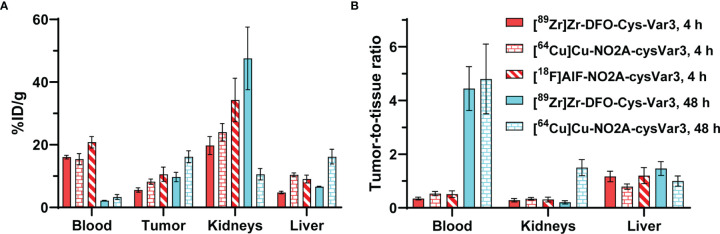
**(A)** Comparison of the biodistribution in the 4T1 tumor model of [^89^Zr]Zr-DFO-Cys-Var3 to [^64^Cu]Cu-NO2A-cysVar3 and [^18^F]AlF-NO2A-cysVar3 from a previously published study (38). **(A)** %ID/g of selected tissues at 4 and 48 hours post-injection of the radiotracers. **(B)** Selected tumor-to-tissue ratios at 4 and 48 hours post-injection of the radiotracers.

Previously, ^18^F-labeled (37), ^99m^Tc-labeled (32), ^64^Cu- and [^18^F]AlF-labeled (38), as well as ^68/67^Ga-labeled (39) pHLIP analogs were investigated in various mouse models. However, the short half-life of the radionuclides or their availability is a limiting factor. The blood clearance of these radiolabeled pHLIP constructs was found to be several hours. For PET imaging, an optimal tumor-to-tissue ratio would be expected after 24 hours or even later. In **Figure 8**, the biodistribution data for [^89^Zr]Zr-DFO-Cys-Var3 to [^64^Cu]Cu-NO2A-cysVar3 and [^18^F]AlF-NO2A-cysVar3, from a previously published study (38), are compared. The half-life of ^18^F (110 minutes) does not allow for awaiting a suitable tumor-to-blood ratio. The four hour timepoint with a tumor-to-blood ratio of 0.51 ± 0.12 corresponds to two half-lives of [^18^F]AlF-NO2A-cysVar3 (25% of activity remained). For [^64^Cu]Cu-NO2A-cysVar3, a suitable tumor-to-blood ratio of 4.8 ± 1.3 was reported for the 48-hour timepoint. However, after 48 hours, only 6% of the short-life ^64^Cu remains (t_1/2_ = 12.7 hours) – challenging for imaging at late timepoints. For this reason, other radionuclides like the widely available ^89^Zr are better suited. Zirconum-89 is a radiometal with a half-life of 3.3 days, which matches the goal of employing long-term imaging using the pHLIP conjugates. ^89^Zr’s relatively low positron energy (E_Avg_=395  keV) allows for high-resolution PET imaging, comparable to fluorine-18 (53). As a further benefit, most medical centers are capable of producing ^89^Zr using low-energy proton-accelerating cyclotrons (14.0–14.5 MeV) and the ^89^Y(p, n)^89^Zr production route (54). However, the release of the ^89^Zr^4+^-ion from the pHLIP compounds should be avoided, since the free radiometal accumulates in the mineral bone, decreasing imaging sensitivity and elevating radiation absorbed doses to the red marrow and skeletal endosteum (55).

This study investigated the influence of different zirconium chelators on the pharmacokinetics of the pHLIP compound. For a higher *in vivo* stability of the peptide, the sequence was built with d-amino acids. Four different chelators were conjugated to the N-terminus of the Var3 and WT pHLIP sequence. The most prominent chelator for the Zr^4+^-ion is DFO, which offers 6 donor atoms for the metal center. The DFO-Zr^4+^-chelate carries a net +1 charge. Three DFO constructs were compared in this study: DFO-Lys-Var3, DFO-Cys-Var3, and DFO-Lys-WT. The cysteine variant used DFO-maleimide for the bifunctional chelator and DFO-NCS for the lysine variant. Despite it being the most commonly used zirconium chelator, reports indicate that DFO can lead to unwanted bone uptake of ^89^Zr due to the chelate’s instability (56). For this reason, we also investigated the chelators DFO* and HOPO, which both form a neutrally-charged chelate with the Zr^4+^-ion. DFO* is structurally similar to DFO but offers an additional hyrdoxamate group, resulting in a total of 8 donor atoms and a more stable chelate (56). The HOPO chelator [3,4,3-(LI-1,2-HOPO)] offers the same amount of donor atoms and was reported to be an excellent alternative to DFO (57). Finally, we investigated DFOsqa, a deferoxamine conjugated to 3,4-diethoxy-3-cyclobutene-1,2-dione (squaric acid diethyl ester). It was reported that squaric acid conjugates enable a fast and simple conjugation to peptides under mild conditions and that the squaric acid might even induce beneficial effects on the pharmacokinetic properties (58). Furthermore, the squaric acid is assumed to coordinate onto the zirconium resulting in a more stable chelate (58).

An efficient single method was developed to radiolabel all six conjugates under mild conditions and in only 30 minutes. The radiolabeling yield was greater than 95%, and no purification steps were necessary. All agents could be easily developed into a clinical kit-like product. The stability of the six [^89^Zr]Zr-pHLIP conjugates was investigated in a serum stability assay and *in vivo* studies. All agents exhibited high stability: lack of degradation and no significant release of free [^89^Zr]Zr^4+^-ions. Biophysical studies confirmed that all agents preserved the ability to interact with the lipid bilayer, driven by a drop in pH. The membrane insertion and folding pK values (in the range of 5.5 to 5.9) and fast membrane-insertion kinetics indicated that agents could potentially sense a low pH (6.0–6.2) at the surface of activated cells within the TME.

The *in vivo* study performed on tumor models in mice, mimicking late-stage prostate and breast cancers, demonstrated a good targeting of the tumors by all Var3-based agents, as well as a high kidney uptake. Tumor targeting ranged from 7–12 %ID/g and kidney uptake increased from 25–83 %ID/g. In the side-by-side comparison study, tumor, kidney, and liver uptake were measured for co-injected radioactive and fluorescent pHLIPs. Fluorescent pHLIP showed significantly lower kidney uptake and slightly higher tumor targeting. Also, regulators of pH [acetazolamide; amiloride and 5-(N,N-dimethyl)amiloride], probenecid, an inhibitor the kidney uptake of organic anions; and chlorthalidone, an inhibitor of sodium reabsorption, failed to reduce the kidney uptake of [^89^Zr]Zr-DFOsqa-Lys-Var3. The results clearly indicate that a high kidney retention is associated with the presence of a metal chelator rather than the pHLIP itself.

[^89^Zr]Zr-DFOsqa-Lys-Var3 resulted in the highest tumor uptake amongst the investigated pHLIP compounds [(12.4 ± 4.7) %ID/g]. However, the kidney uptake was (82.5 ± 14.2) %ID/g, and the attempts to reduce this value by employing “kidney blocking agents” did not show the expected response. For this reason, we selected DFO-Cys-Var3 for a possible clinical translation, which exhibits high tumor targeting [(7.3 ± 2.9) %ID/g] and a moderate kidney uptake [(25.7 ± 3.0) %ID/g)]. The serial biodistribution study (4, 24, 48, 72, and 120 hours) confirmed the results of the imaging study using the 4T1 tumor model. Moderate blood clearance of [^89^Zr]Zr-DFO-Cys-Var3 was observed. The blood-activity concentration at 24 hours was (5.8 ± 0.5) %ID/g; the biological blood clearance half-life was (16.0 ± 0.4) hours. The biological half-life for total body excretion was (415 ± 10) hours; minimal uptake was evident in the contents of the bladder or gastrointestinal tract on the PET images. Notably, in the PET images, kidney uptake was primarily localized to the cortex and minimal in the renal medulla or pelvis. An optimal tumor uptake was detected for the 48-hour timepoint (9.7 ± 1.7) %ID/g. Slight differences for the tumor- and kidney-uptakes were determined between the two tumor models and among different sizes of tumors. The differences can be related to the aggressiveness and acidity of the tumor. Human reference dosimetry estimates are required usage approval of [^89^Zr]Zr-DFO-Cys-Var3 in investigational new drug studies and for documentation. In terms of the estimated human dosimetry, the effective dose coefficient was in line with full-length ^89^Zr-labeled monoclonal antibodies (59). The major difference in dosimetry was that, for [^89^Zr]Zr-DFO-Cys-Var3, the kidneys were the critical organ; however, no kidney toxicity is expected. Our preclinical biodistribution results, together with the human dosimetry estimates, suggest that [^89^Zr]Zr-DFO-Cys-Var3 will be safe and effective at administered activities required to obtain diagnostic quality PET images in human patients.

## Conclusion

Detailed investigation of the pharmacokinetics of the Var3 PET agents within the TME performed on tumor sections revealed that the agents stain the entire tumor mass and highlighted areas of acidification within the tumor–stroma interface. It was previously shown that targeting by fluorescent pHLIPs is not restricted to hypoxic areas. As sensors of the cell surface acidity, pHLIP constructs target the stroma (50), specifically metabolically active macrophages within the environment surrounding the cancer cells (28). In the era of immuno-oncology, this is an important finding toward improving therapy outcomes. An acidic TME created by both cancer cells and TAMs inhibits the presence of T-cells within the TME and suppresses the cytotoxic functions of T-cells (60). Acidity facilitates tumor growth, leads to drug resistance, and promotes immuno-suppression (60, 61). Thus, the development of novel imaging probes for tumor acidity has a high significance. Such probes would allow clinicians to predict and monitor the outcome of immunotherapies. pHLIP agents are very well suited for this task, but they exhibit slow blood clearance and tumor targeting, as established in preclinical and clinical studies. [^89^Zr]Zr-DFO-Var3, containing the long-lived ^89^Zr isotope, could be imaged for several days after administration, suggesting its potential for clinical translation for the identification of optimal imaging timepoints and, eventually, for supporting immuno-oncology therapeutics.

## Data Availability Statement

The original contributions presented in the study are included in the article/**Supplementary Material**. Further inquiries can be directed to the corresponding author.

## Ethics Statement

The animal study was reviewed and approved by Institutional Animal Care & Use Committee (IACUC) at Memorial Sloan Kettering (MSK).

## Author Contributions

DB, YKR, and JSL conceived and designed this research. DB was involved in the chemistry, radiochemistry, and imaging. HV, AW, OAA, and YKR were involved in the biophysical characterization. DB and ZS were involved in the preparation of radiopharmaceuticals in this study. DB, ZS, and SK took part in the animal experiments. LMC calculated the dosimetry estimations. All authors contributed to the article and approved the submitted version.

## Funding

This work was supported by NIH R01 GM073857 (OAA, YKR), NIH F32 EB025050 (LMC), and NCI R35 CA232130 (JSL).

Technical services provided by the MSK Small-Animal Imaging Core Facility, supported in part by NIH Cancer Center Support Grant P30 CA008748, NIH Shared Instrumentation Grants S10 RR02892-01 and S10 OD016207-01.

## Conflict of Interest

OAA, YKR and JSL are founders of pHLIP, Inc. They have shares in the company, but the company did not fund any part of the work reported in this paper, which was carried out in their academic laboratories.

The remaining authors declare that the research was conducted in the absence of any commercial or financial relationships that could be construed as a potential conflict of interest.

## Publisher’s Note

All claims expressed in this article are solely those of the authors and do not necessarily represent those of their affiliated organizations, or those of the publisher, the editors and the reviewers. Any product that may be evaluated in this article, or claim that may be made by its manufacturer, is not guaranteed or endorsed by the publisher.

## Abbreviations

**%ID/g**, mean percent injected dose per gram tissue; **CA IX**, Carbonic Anhydrase IX; **CD**, circular dichroism; **Cys**, cysteine, **DFO*-NCS**, 5,11,16,22-Tetraazahexacosanediamide, N1-[5-(acetylhydroxyamino)pentyl]-N26,5,16-trihydroxy-N26-[5-[[[(4-isothiocyanatophenyl)amino]thioxomethyl]amino]pentyl]-4,12,15,23-tetraoxo-; **DFO-maleimide**, N-(3,11,14,22,25,33-hexaoxo-4,10,15,21,26,32-hexaaza-10,21,32-trihydroxytetratriacontane)maleimide; **DFO-NCS**, 1-(4-isothiocyanatophenyl)-3-[6,17-dihydroxy-7,10,18,21-tetraoxo-27-(N-acetylhydroxylamino)-6,11,17, 22-tetraazaheptaeicosine] thiourea; **DFOsqa**, deferoxamine squaramide ethyl ester; **DMEM**, Dulbecco’s Modified Eagle Medium; **DMSO**, dimethyl sulfoxide; **EDTA**, ethylenediaminetetraacetic acid; **HEPES**, 4-(2-hydroxyethyl)-1-piperazineethanesulfonic acid; **HOPO**, 3,4,3-(LI-1,2-HOPO); **HPLC**, high-performance liquid chromatography; **IO**, immuno oncology; **iTLC**, instant thin layer chromatography; **Lys**, lysine; **MIP**, maximum intensity projection; **PBS**, phosphate-buffered saline; **PET**, positron emission tomography; **pHLIP**, pH-Low Insertion Peptide; **POPC**, 1-palmitoyl-2-oleoyl-*sn*-glycero-3-phosphocholine; **RPMI**, RPMI 1640 medium; **SUV**, standardized uptake value, normalized by body mass; **TAMs**, tumor-associated macrophages; **TME**, tumor microenvironment; **Trp**, tryptophan
