# Supplementary material for: PET Imaging of Acidic Tumor Environment With 89Zr-labeled pHLIP Probes
Source: Front Oncol. 2022 May 19;12:882541. doi: 10.3389/fonc.2022.882541 (PMC9160799; doi:10.3389/fonc.2022.882541)
Supplement: Supplementary file 1 [file DataSheet_1.pdf]

Supplementary Material

Supplementary Material

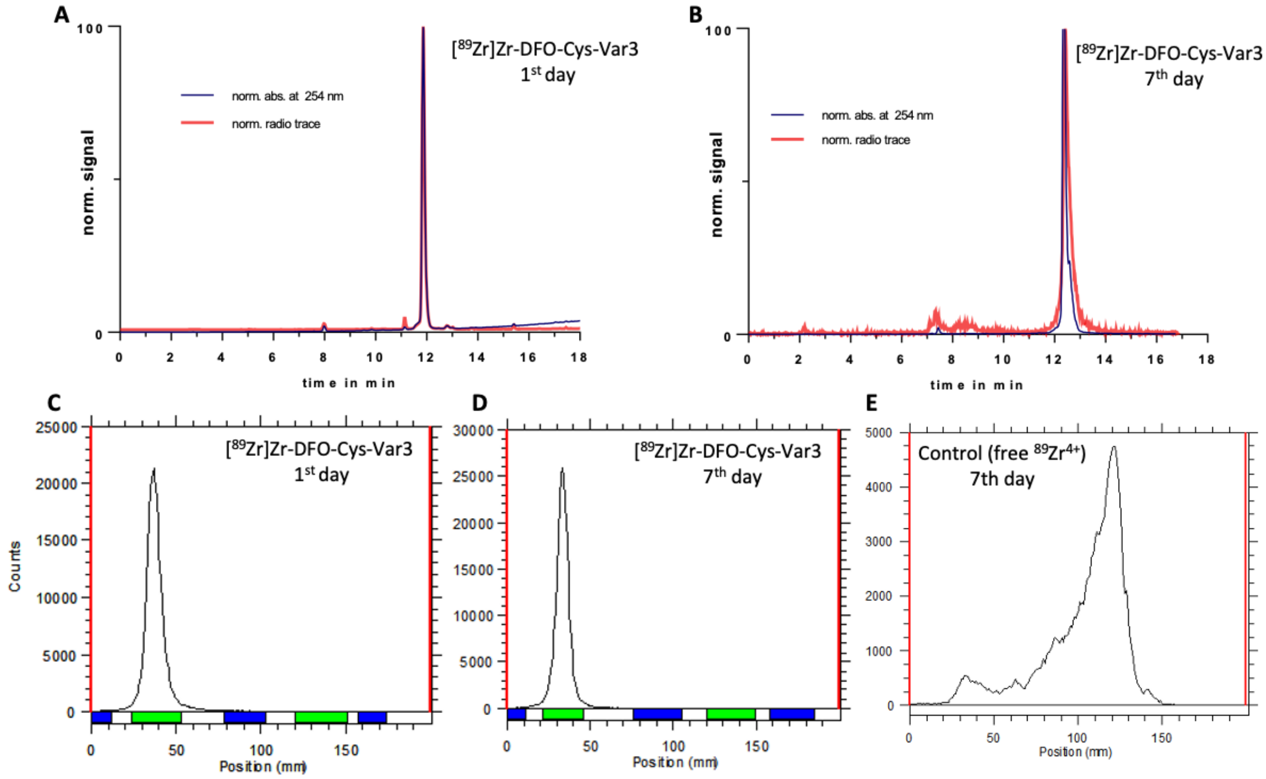

Figure S1. Top: HPLC of [^{89}Zr]Zr-DFO-Cys-Var3, normalized radio trace in red, and normalized 254-nm absorbance in blue, **(A)** after radiolabeling, and (**B)** after an incubation in human serum for 7 days (radiochemical purity > 90%); **(C)** radio iTLC of [^{89}Zr]Zr-DFO-Cys-Var3 after radiolabeling; **(D)** radio iTLC of [^{89}Zr]Zr-DFO-Cys-Var3 after an incubation in human serum for 7 days (no release of $^{89}Zr^{4+}$ was detected), and **(E)** radio iTLC of [^{89}Zr]zirconium oxalate after an incubation of 7 days in human serum as control.

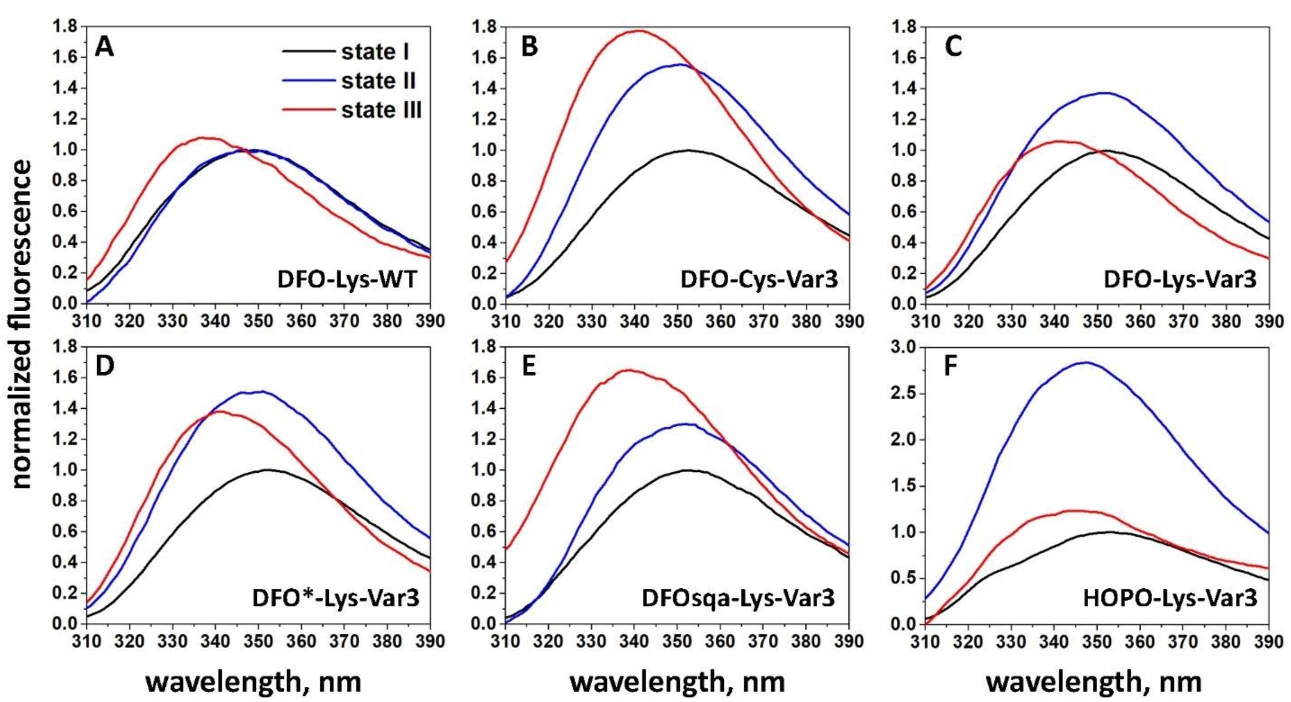

Figure S2. Normalized tryptophan fluorescence is shown for pHLIP agents: in aqueous solution at pH 8 (black lines, state I), at pH 8 in the presence of POPC liposomes (blue lines, state II) and at pH 4 in the presence of POPC liposomes (red lines, state III).

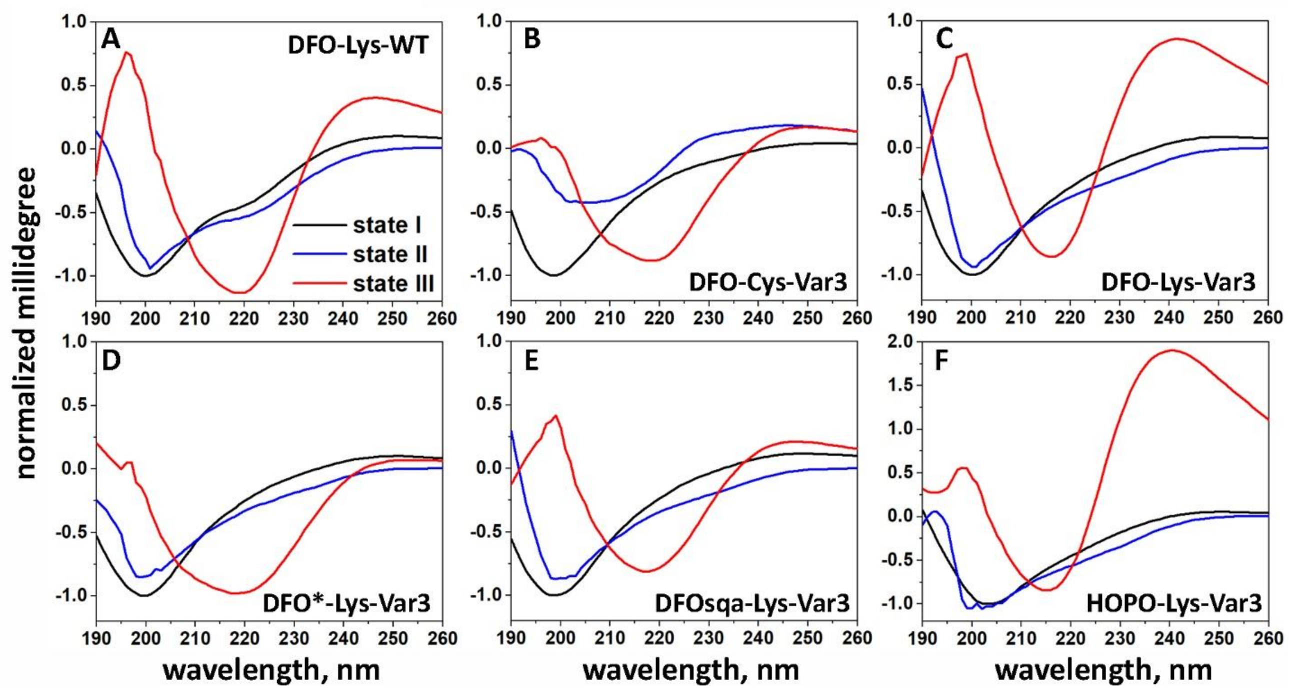

Figure S3. Normalized CD is shown for pHLIP agents: in aqueous solution at pH 8 (black lines, state I), at pH 8 in the presence of POPC liposomes (blue lines, state II) and at pH 4 in the presence of POPC liposomes (red lines, state III).

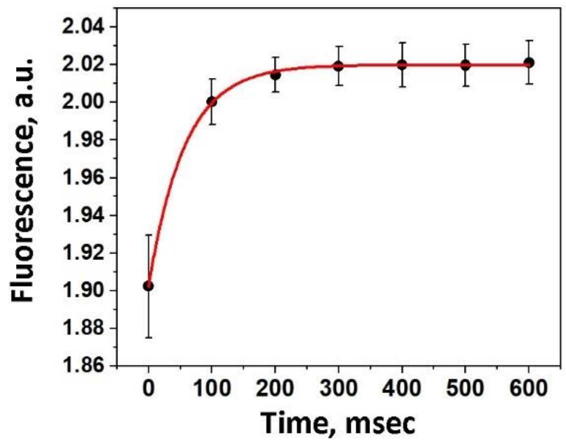

Figures S4. Average fluorescence intensity reflecting insertion of DFO-Cys-Var3 into the lipid bilayer triggered by drop of pH from pH 8 to pH 4. The fluorescence signal was excited at 295 nm and measured via 320 nm cut off filter. The experimental data (black circles) and curve of single exponential fit (red line) are shown.

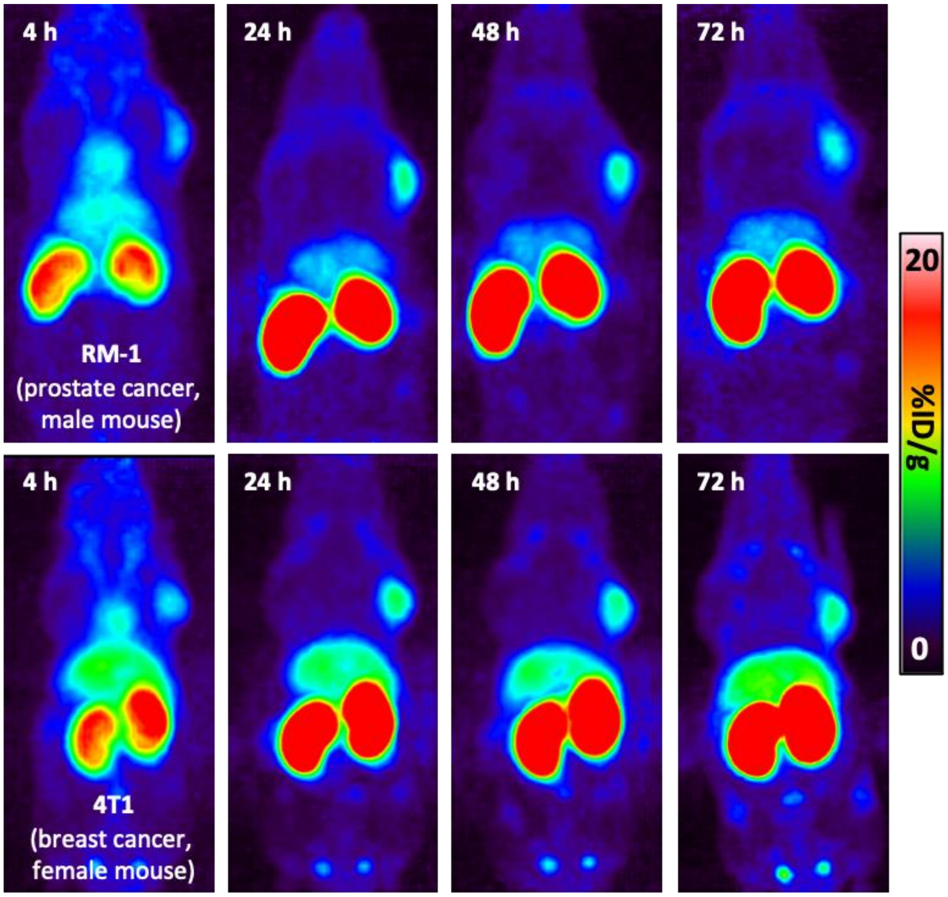

Figure S5. Coronal MIP PET images at 4, 24, 48, and 72 hours of male athymic nude mice bearing subcutaneous RM-1 tumor allografts (top) and female athymic nude mice bearing subcutaneous 4T1 tumor allografts (bottom) on the right shoulder, administered with 7.4 MBq/200 µCi of [^{89}Zr]Zr-DFOsqa-Lys-Var3 (1.2 nmol pHLIP).

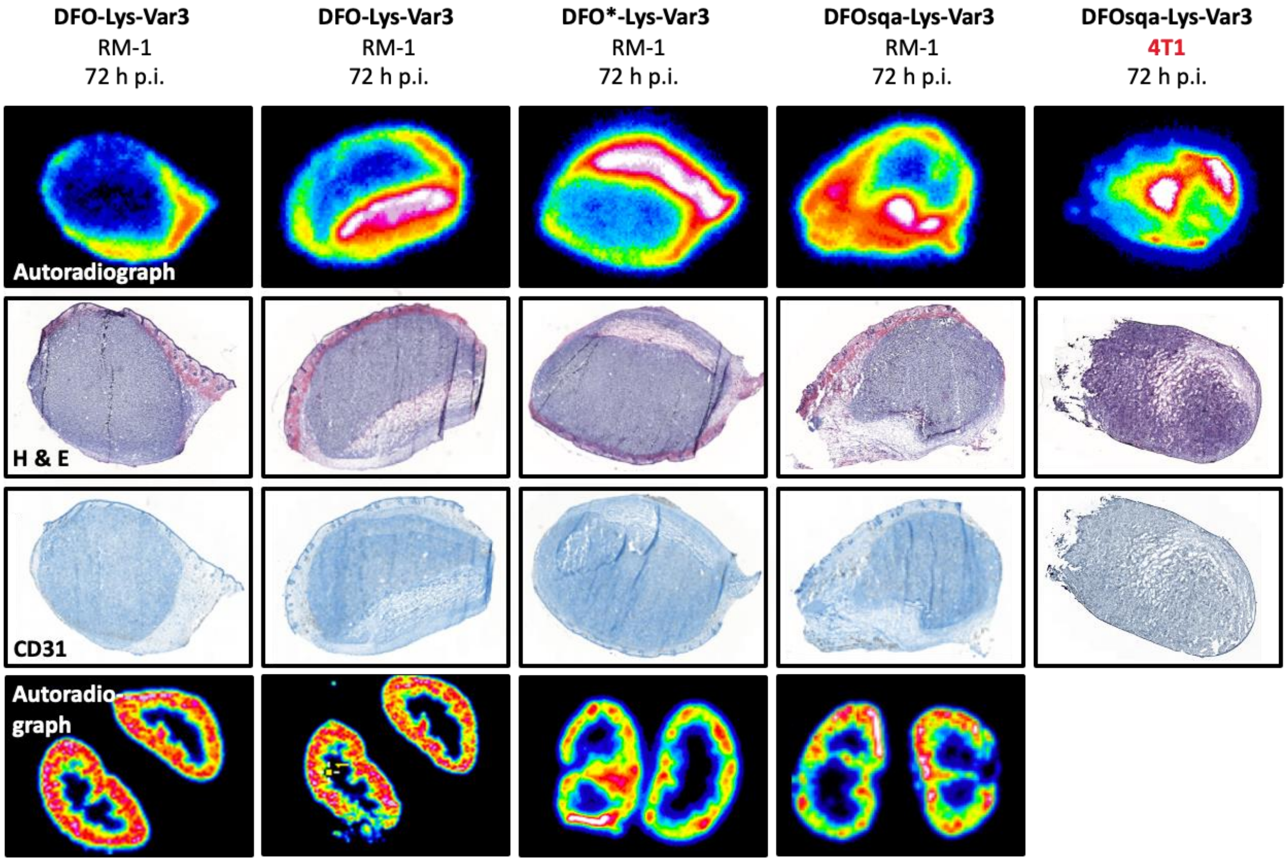

Figure S6. Slices of various resected tumors (row 1-3), 72 hours after the injection of the ^{89}Zr-labeled pHLIP compound. The images represent the autoradiographs (visualizing the radiotracer distribution, 1st row), H&E staining (visualizing cells and cell membranes, 2nd row), and CD31 staining (visualizing murine blood vessels, 3rd row) of contiguous tumor sections of 10 µm thickness. The pHLIP agents are present in the entire tumor mass, and the areas of the highest activity are overlapping with the tumor's stroma. The 4th row represents the autoradiographs of 10-µm kidney sections; the pHLIP agents are present primary in the kidney's cortex. The autoradiographs are not scaled or calibrated and cannot be compared directly.

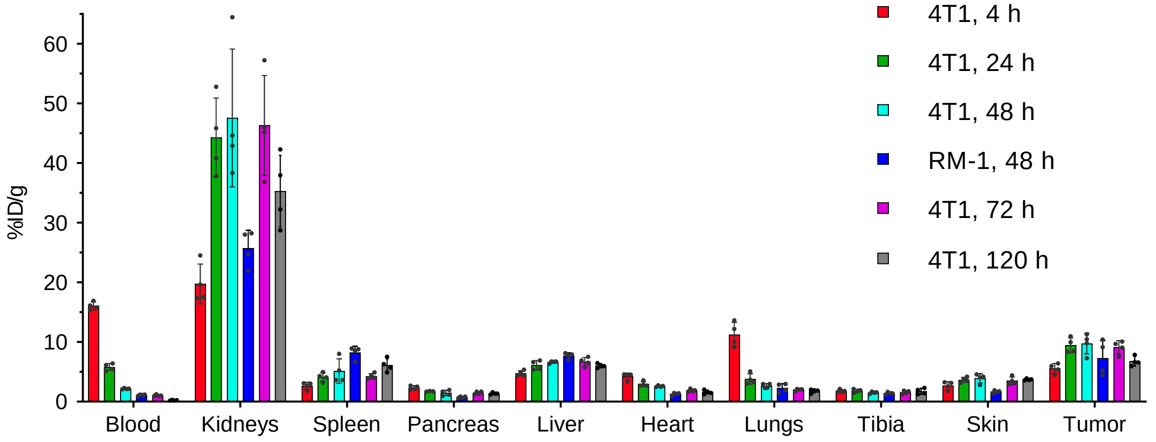

Figure S7. Biodistribution data of the DFO-Cys-Var3 pHLIP at 4, 24, 48, 72, and 120 hours (p.i.) evaluated in female athymic nude mice using the 4T1 tumor model (n=4). The data in blue represents the 48-hours timepoint in male athymic nude mice and the RM-1 tumor model.

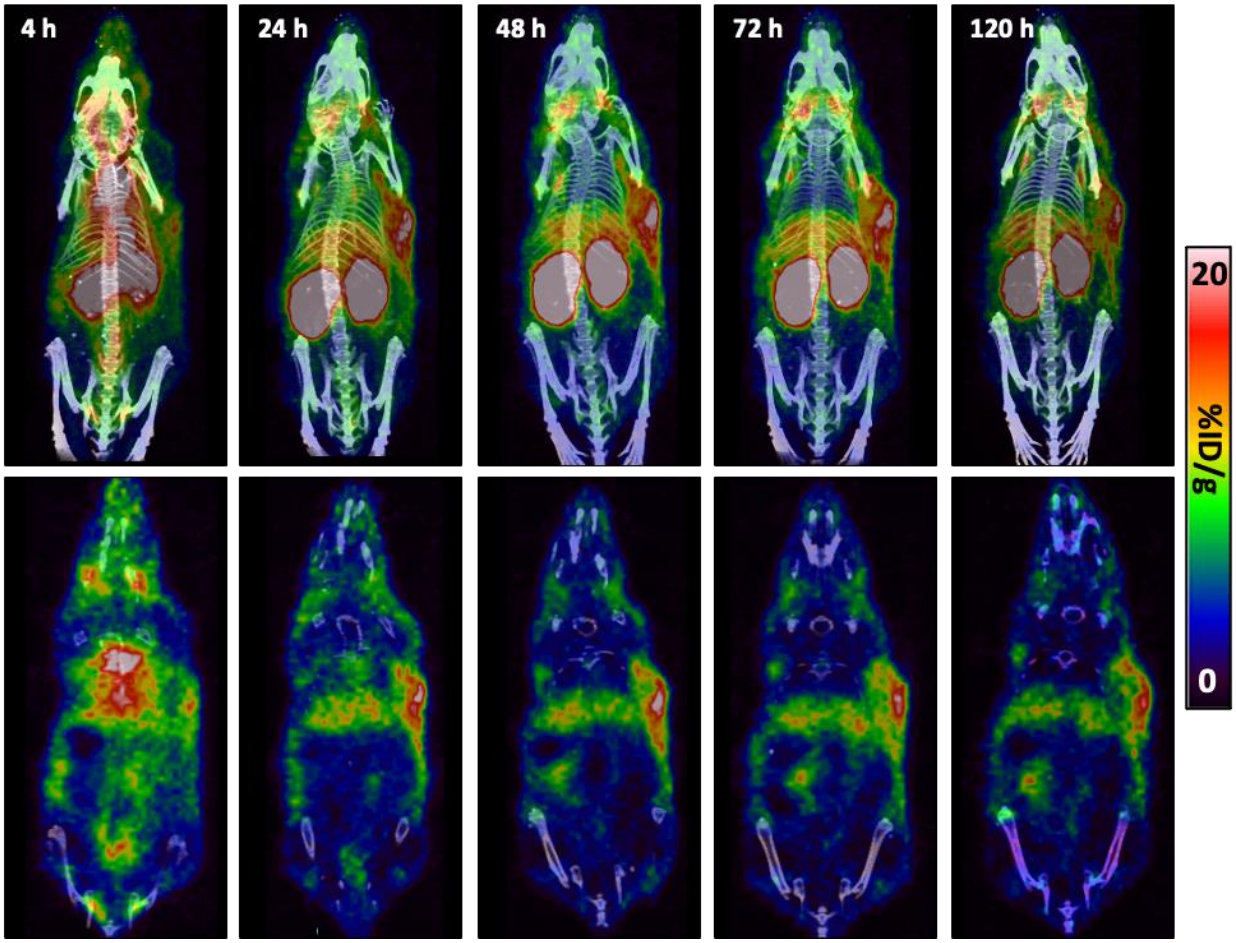

Figure S8. Coronal MIP PET-CT images (top) and coronal 0.8 mm-slices of the tumor hotspots (bottom) at 4, 24, 48, 72, and 120 hours of female athymic nude mice bearing subcutaneous 4T1 tumor allografts on the right shoulder, administered with 7.4 MBq/200 µCi of [^{89}Zr]Zr-DFO-Cys-Var3 (1.2 nmol pHLIP).

Table S1. Overview of the animal experiments. Each mouse received 7.4 MBq/200 µCi of [^{89}Zr]Zr-pHLIP (1.2 nmol) via tail vein injection.

pHLIP compound	Tumor model, mouse strain	Biodistribution study	Imaging	Number of mice
**DFO-Lys-WT**	RM-1, male athymic nude mice	48-hour timepoint (n=4)	PET at 4-, 24-, 48-, and 72-hour timepoint	5*****
**DFO-Cys-Var3**	RM-1, male athymic nude mice	48-hour timepoint (n=4)	PET at 4-, 24-, 48-, and 72-hour timepoint	5*****
	4T1, female athymic nude mice	4-, 24-, 48-, 72-, and 120-hour timepoint (n=4)	4-, 24-, 48-, 72-, and 120-hour timepoint	20
**DFO-Lys-Var3**	RM-1, male athymic nude mice	48-hour timepoint (n=4)	PET at 4-, 24-, 48-, and 72-hour timepoint	5*****
**DFO*-Lys-Var3**	RM-1, male athymic nude mice	48-hour timepoint (n=4)	PET at 4-, 24-, 48-, and 72-hour timepoint	5*****
**HOPO-Lys-Var3**	RM-1, male athymic nude mice	48-hour timepoint (n=4)	PET at 4-, 24-, 48-, and 72-hour timepoint	5*****
**DFOsqa-Lys-Var3**	RM-1, male athymic nude mice	48-hour timepoint (n=4)	PET at 4-, 24-, 48-, and 72-hour timepoint	5*****
		48-hour timepoint for fluorescent and Cherenkov imaging of selected organs (n=5)		5
	4T1, female athymic nude mice	48-hour timepoint (n=4)	PET at 4-, 24-, 48-, and 72-hour timepoint	5*****
	healthy female SCID mice (kidney-blocking study)	-	24-hour timepoint	22

****** The kidneys and tumors of theses mice were used for ex vivo autoradiography, staining and microscopy*

Table S2. Biodistribution data (in %ID/g, mean ± S.D.) of the DFO-Cys-Var3 pHLIP at 4, 24, 48, 72, and 120 hours (p.i.) evaluated in female athymic nude mice using the 4T1 tumor model (n=4). The last column represents the data for of the 48-hours timepoint in male athymic nude mice and the RM-1 tumor model (n=4).

**Tissue (n=4)**	**4 h (4T1)**	**24 h (4T1)**	**48 h (4T1)**	**72 h (4T1)**	**120 h (4T1)**	**48 h (RM-1)**
**Blood**	16.1 ± 0.6	5.8 ± 0.5	2.2 ± 0.1	1.0 ± 0.1	0.2 ± 0.05	1.1 ± 0.1
**Kidneys**	**19.8 ± 3.3**	**44.3 ± 6.5**	**47.6 ± 11.5**	**46.3 ± 8.3**	**35.3 ± 5.9**	**25.7 ± 3.0**
**Spleen**	2.7 ± 0.5	4.1 ± 0.7	5.1 ± 2.0	4.3 ± 0.4	6.1 ± 1.0	8.2 ± 1.1
**Pancreas**	2.3 ± 0.3	1.8 ± 0.2	1.5 ± 0.4	1.5 ± 0.2	1.4 ± 0.1	0.7 ± 0.1
**Liver**	4.8 ± 0.4	6.1 ± 0.7	6.6 ± 0.1	6.7 ± 0.7	6 ± 0.3	7.7 ± 0.5
**Heart**	4.2 ± 0.5	3.0 ± 0.4	2.6 ± 0.1	1.9 ± 0.2	1.6 ± 0.3	1.3 ± 0.4
**Lungs**	11.2 ± 2.0	3.8 ± 0.9	2.7 ± 0.3	2.0 ± 0.1	1.8 ± 0.3	2.3 ± 0.2
**Tibia**	1.8 ± 0.2	1.8 ± 0.06	1.5 ± 0.4	1.6 ± 0.2	1.7 ± 0.5	1.4 ± 0.1
**Skin**	2.7 ± 0.6	3.7 ± 0.4	3.9 ± 0.7	3.5 ± 0.6	3.7 ± 0.1	1.7 ± 0.2
**Tumor**	**5.6 ± 0.8**	**9.4 ± 1.2**	**9.7 ± 1.7**	**9.1 ± 1.0**	**6.8 ± 0.7**	**7.3 ± 2.9**

Table S3. Human dose estimates extrapolated from murine biodistribution. $F_{\gamma,x}$: fractional contribution to the absorbed dose by photons (x- and γ-rays). $F_{\beta+,e-}$: fractional contribution to the absorbed dose by electrons (positrons and monoenergetic electrons).

	Adult male			Adult female		
**Target organs**	**Absorbed dose [mGy/MBq]**	$F_{\gamma,x}$	$F_{\beta+,e-}$	**Absorbed dose [mGy/MBq]**	$F_{\gamma,x}$	$F_{\beta+,e-}$
**Adipose tissue**	3.60E-01 ± 4.4E-03	79%	21%	4.01E-01 ± 4.7E-03	78%	23%
**Adrenals**	9.34E-01 ± 4.7E-02	90%	10%	9.91E-01 ± 3.8E-02	89%	11%
**Bone - endosteal cells**	4.67E-01 ± 4.1E-03	79%	21%	5.42E-01 ± 4.7E-03	80%	20%
**Bone marrow - red (active)**	4.91E-01 ± 5.1E-03	81%	19%	5.64E-01 ± 6.0E-03	81%	19%
**Brain**	3.26E-01 ± 3.9E-03	78%	23%	3.82E-01 ± 4.6E-03	76%	24%
**Breast tissue**	3.09E-01 ± 3.4E-03	77%	23%	3.84E-01 ± 3.9E-03	77%	23%
**Bronchial basal cells**	4.88E-01 ± 1.2E-02	71%	29%	6.00E-01 ± 1.4E-02	72%	28%
**Bronchial secretory cells**	4.87E-01 ± 1.2E-02	71%	29%	5.99E-01 ± 1.4E-02	72%	28%
**Bronchiolar secretory cells**	4.55E-01 ± 9.1E-03	79%	21%	5.05E-01 ± 9.5E-03	81%	19%
**Colon - ICRP133**	4.43E-01 ± 7.1E-03	87%	13%	4.99E-01 ± 6.6E-03	86%	14%
**Colon - left**	4.25E-01 ± 7.7E-03	86%	13%	4.80E-01 ± 5.9E-03	85%	15%
**Colon - rectosigmoid**	4.12E-01 ± 4.3E-03	86%	14%	4.94E-01 ± 5.1E-03	86%	14%
**Colon - right**	4.78E-01 ± 9.2E-03	88%	12%	5.20E-01 ± 9.7E-03	86%	14%
**Esophagus**	4.82E-01 ± 4.8E-03	88%	12%	5.47E-01 ± 5.1E-03	86%	14%
**ET1 airway basal cells**	1.87E-01 ± 1.5E-03	83%	16%	2.73E-01 ± 2.5E-03	86%	14%
**ET2 airway basal cells**	3.01E-01 ± 2.6E-03	89%	11%	3.82E-01 ± 3.0E-03	90%	10%
**Extrathoracic region - ICRP133**	2.96E-01 ± 2.5E-03	89%	11%	3.78E-01 ± 3.0E-03	90%	10%
**Eye lens**	2.17E-01 ± 2.0E-03	74%	26%	2.61E-01 ± 2.4E-03	74%	26%
**Gallbladder wall**	6.57E-01 ± 1.5E-02	89%	11%	8.62E-01 ± 2.1E-02	89%	11%
**Heart wall**	4.34E-01 ± 5.2E-03	89%	11%	4.96E-01 ± 5.4E-03	88%	12%
**Kidneys**	1.83E+00 ± 1.1E-01	57%	43%	2.18E+00 ± 1.4E-01	56%	44%
**Liver**	6.66E-01 ± 1.4E-02	80%	20%	8.05E-01 ± 1.2E-02	78%	22%
**Lung - ICRP133**	4.39E-01 ± 8.2E-03	82%	18%	5.00E-01 ± 9.2E-03	82%	19%
**Lungs (AI)**	4.39E-01 ± 8.2E-03	82%	18%	5.00E-01 ± 9.2E-03	81%	19%
**Lymph nodes - extrathoracic**	3.84E-01 ± 3.4E-03	80%	20%	4.92E-01 ± 3.9E-03	80%	20%
**Lymph nodes - systemic**	4.64E-01 ± 5.4E-03	83%	17%	5.54E-01 ± 7.9E-03	83%	17%
**Lymph nodes - thoracic**	4.46E-01 ± 4.5E-03	84%	17%	5.25E-01 ± 5.7E-03	81%	19%
**Lymphatic nodes - ICRP133**	4.55E-01 ± 4.9E-03	83%	17%	5.46E-01 ± 7.0E-03	83%	17%
**Muscle**	3.63E-01 ± 4.5E-03	80%	20%	4.45E-01 ± 5.2E-03	80%	20%
**Oral mucosa**	4.29E-01 ± 3.4E-03	70%	30%	5.03E-01 ± 3.7E-03	70%	30%
**Ovaries**	--	--	--	5.51E-01 ± 5.5E-03	83%	17%
**Pancreas**	5.71E-01 ± 1.3E-02	93%	7%	7.23E-01 ± 2.6E-02	93%	7%
**Pituitary gland**	3.59E-01 ± 3.7E-03	80%	20%	4.60E-01 ± 4.4E-03	79%	20%
**Prostate**	4.61E-01 ± 5.0E-03	84%	16%	--	--	--
**Salivary glands**	3.17E-01 ± 2.9E-03	77%	23%	4.02E-01 ± 3.4E-03	78%	22%
**Skin**	2.54E-01 ± 2.7E-03	75%	25%	2.97E-01 ± 3.2E-03	74%	26%
**Small intestine**	4.66E-01 ± 7.8E-03	89%	11%	5.63E-01 ± 1.1E-02	89%	11%
**Spleen**	5.64E-01 ± 2.4E-02	83%	17%	6.66E-01 ± 2.9E-02	82%	18%
**Stomach**	4.60E-01 ± 7.5E-03	89%	11%	5.74E-01 ± 1.3E-02	89%	11%
**Testes**	3.55E-01 ± 4.1E-03	80%	20%	--	--	--
**Thymus**	4.15E-01 ± 3.9E-03	82%	18%	4.95E-01 ± 4.7E-03	82%	18%
**Thyroid**	3.83E-01 ± 3.6E-03	81%	19%	4.54E-01 ± 4.2E-03	81%	20%
**Tongue**	4.02E-01 ± 3.3E-03	75%	25%	4.69E-01 ± 3.6E-03	76%	24%
**Tonsils**	3.81E-01 ± 3.3E-03	81%	19%	4.49E-01 ± 3.6E-03	80%	20%
**Ureters**	5.82E-01 ± 1.2E-02	87%	13%	6.66E-01 ± 1.4E-02	86%	14%
**Urinary bladder wall**	4.53E-01 ± 4.4E-03	84%	16%	4.92E-01 ± 4.4E-03	82%	18%
**Uterus**	--	--	--	5.50E-01 ± 5.2E-03	84%	16%
**Whole body target**	3.85E-01 ± 4.5E-03	79%	21%	4.49E-01 ± 5.0E-03	78%	22%
**Effective dose [mSv/MBq]**	4.96E-01 ± 2.5E-03	--	--	4.96E-01 ± 2.5E-03	--	--

Table S4. The position of maximum (**λ_{max}**) of tryptophan fluorescence spectra of pHLIP agents in state I, II and III, the midpoint of transition (**pK**) and cooperativity (***n***) of the selected agent's insertion into the lipid bilayer of membrane of POPC liposomes monitored by the changes of the maximum of tryptophan fluorescence and CD in the result of pH drop from pH 8 to pH 3. The fluorescence spectra are shown in Figure S1 and Figure S4 and the pH-dependency plots are shown in Figure 3.

**pHLIP construct**		**λ_{max}, nm**			**pK and *n***	
		**state I**	**state II**	**state III**	**Fluor.**	**CD**
**with Zr**	DFO-Lys-WT	347.2	346.8	338.9	-	-
	DFO-Cys-Var3	352.4	348.8	340.1	5.62 ± 0.02<br>0.92 ± 0.04	5.62 ± 0.01<br>1.57 ± 0.04
	DFO-Lys-Var3	352.2	349.7	341.6	5.93 ± 0.03<br>0.92 ± 0.06	5.76 ± 0.03<br>1.09 ± 0.07
	DFO*-Lys-Var3	352.0	348.2	341.4	5.87 ± 0.07<br>0.79 ± 0.11	5.47 ± 0.01<br>1.46 ± 0.05
	DFOsqa-Lys-Var3	352.4	352.4	340.2	5.77 ± 0.09<br>0.71 ± 0.11	5.75 ± 0.02<br>1.22 ± 0.05
	HOPO-Lys-Var3	352.6	347.2	346.1	-	-
**without Zr**	DFO*-Lys-Var3	351.9	349.3	341.4	-	-
	DFOsqa-Lys-Var3	352.4	351.5	342.3	-	-

Table S5. Biodistribution data (in %ID/g, mean ± S.D.) of the six investigated pHLIP constructs at the 48-hours timepoint (p.i.) evaluated in male athymic nude mice using the RM-1 tumor model (n=4). The last column represents the data for DFOsqa-Lys-Var3 (48-hours timepoint) in female athymic nude mice and the 4T1 tumor model (n=4).

Tissue (n=4)	DFO-Lys-WT (RM-1)	DFO-Cys-Var3 (RM-1)	DFO-Lys-Var3 (RM-1)	DFO*-Lys-Var3 (RM-1)	HOPO-Lys-Var3 (RM-1)	DFOsqa-Lys-Var3 (RM-1)	DFOsqa-Lys-Var3 (4T1)
**Blood**	0.1 ± 0.03	1.1 ± 0.1	0.5 ± 0.1	0.4 ± 0.09	0.2 ± 0.03	0.3 ± 0.03	0.4 ± 0.01
**Kidneys**	**14.2 ± 1.2**	**25.7 ± 3.0**	**27.9 ± 3.6**	**57.5 ± 9.6**	**39.8 ± 0.7**	**82.5 ± 14.2**	**82.5 ± 10.9**
**Spleen**	25.8 ± 5.5	8.2 ± 1.1	7.2 ± 0.7	4.4 ± 0.9	6.4 ± 0.3	6.4 ± 1.4	6.9 ± 0.6
**Pancreas**	0.9 ± 0.4	0.7 ± 0.1	1.0 ± 0.2	1.1 ± 0.4	1.2 ± 0.2	1.2 ± 0.1	0.8 ± 0.1
**Liver**	14.8 ± 4.7	7.7 ± 0.5	8.7 ± 0.8	6.7 ± 1.2	8.4 ± 0.8	10.8 ± 2.1	11.2 ± 1.0
**Heart**	0.7 ± 0.1	1.3 ± 0.4	1.3 ± 0.03	1.5 ± 0.3	1.2 ± 0.1	1.6 ± 0.1	1.3 ± 0.06
**Lungs**	11.5 ± 0.4	2.3 ± 0.2	2.0 ± 0.6	1.7 ± 0.2	1.3 ± 0.08	2.5 ± 0.6	1.4 ± 0.2
**Muscle**	0.3 ± 0.08	0.6 ± 0.7	0.5 ± 0.1	0.8 ± 0.2	0.5 ± 0.03	0.7 ± 0.09	0.5 ± 0.04
**Tibia**	2.3 ± 0.5	1.4 ± 0.1	1.4 ± 0.2	1.1 ± 0.5	1.4 ± 0.3	1.6 ± 0.1	1.5 ± 0.2
**Skin**	1.5 ± 0.2	1.7 ± 0.2	1.7 ± 0.3	2.4 ± 0.5	2.0 ± 0.1	2.1 ± 0.07	2.6 ± 0.4
**Tumor**	**1.6 ± 0.1**	**7.3 ± 2.9**	**6.9 ± 1.9**	**10.2 ± 5.6**	**5.2 ± 1.3**	**12.4 ± 4.7**	**10.2 ± 1.2**
**Brain**	0.03 ± 0.03	0.08 ± 0.01	0.06 ± 0.01	0.07 ± 0.02	0.03 ± 0.01	0.06 ± 0.02	0.04 ± 0.01

Table S6. Summary of the kidney-blocking study. Healthy female SCID mice (2 mice per experiment/ dosage + 2 control mice without treatment, total of 22 mice) received a blocking agent intravenously 30 minutes prior to the injection of 7.4 MBq/200 µCi [^{89}Zr]Zr-DFOsqa-Lys-Var3 (1.2 nmol pHLIP). The drug dose was calculated as weight of drug (mg) per weight of mouse (kg). A potential kidney clearance (uptake < 80 %ID/g) was measured by using *in vivo* PET imaging and comparing the calibrated images to the control mice (24 hours post injection). No drug led to a significant reduction of the kidney uptake.

drug (IV 30 min before [^{89}Zr]Zr-pHLIP)	dose [mg/kg] (n=2)	effect on kidney uptake	mechanism of action
**amiloride**	1 and 4	not significant	blocks epithelial sodium channel (ENaC); promotes loss of sodium and water from the body; acts as a diuretic
**5-(N,N-dimethyl) amiloride**	2 and 4	not significant	more potent amiloride analog
**probenecid**	50 and 100	not significant	gets reabsorbed in the distal tubule; interferes with the kidneys' organic anion transporter; blocks the uptake of organic anions
**chlorthalidone**	10 and 20	not significant	inhibits sodium reabsorption at the level of the distal convoluted tubule via inhibition of the Na/Cl symporter; acts as a diuretic
**acetazolamide**	20 and 40	not significant	carbonic anhydrase inhibitor; urinary Na^+ and HCO_3^- levels are increased – serum levels decreased
